# Voltammetric analysis of pholcodine on graphene-modified GNPs/PTs with green assessment

**DOI:** 10.1186/s13065-024-01146-x

**Published:** 2024-03-06

**Authors:** Nahla A. Abdelshafi, Hany W. Darwish, Ashwag S. Alanazi, Ibrahim A. Naguib, Hadeer H. Elkhouly, Nehal S. Khodary, Ekram H. Mohamed

**Affiliations:** 1 Department of Pharmaceutical Analytical Chemistry, School of Pharmacy, Badr University in Cairo, Badr City, Cairo, 11829 Egypt; 2 Department of Pharmaceutical Chemistry, College of Pharmacy, King Saud University, 11451 Riyadh, Saudi Arabia; 3 Department of Pharmaceutical Sciences, College of Pharmacy, Princess Nourah Bint Abdulrahman University, 11671 Riyadh, Saudi Arabia; 4 Department of Pharmaceutical Chemistry, College of Pharmacy, Taif University, P.O. Box 11099, 21944 Taif, Saudi Arabia; 5 School of Pharmacy, Badr University in Cairo, Badr City, Cairo, 11829 Egypt; 6 Pharmaceutical Chemistry Department, Faculty of Pharmacy, The British University in Egypt, El Sherouk City, Cairo, 11837 Egypt

**Keywords:** Graphene nanoplatelets, Polythiophene, Pholcodine, Square wave voltammetry, Green assessment

## Abstract

Pholcodine, an anti-tussive medication widely used as an over-the-counter, OTC drug, has recently faced restrictions in several countries. This paper presents a sensitive electrochemical approach for pholcodine detection. The electrochemical method involved fabricating a graphene nanoplatelets electrode, incorporating polythiophene nanospheres polymer to promote electron transfer and increase the activated surface area. Characterization of the fabricated electrode was performed using transmission electron microscopy, ATR-Fourier-transform infrared spectroscopy, X-ray crystallography, X-ray photoelectron spectroscopy, and electrochemical impedance spectroscopy. The electrochemical behavior of pholcodine with the fabricated electrode was investigated using cyclic voltammetry, chronoamperometry, square wave voltammetry (SWV), and differential pulse voltammetry (DPV). The developed electrode led to a linear response for pholcodine ranging from 10 to 45 mg/L with detection limits of 1.41 and 1.51 mg/mL for SWV and DPV, respectively and quantification limits of 4.27 and 4.57 mg/L for SWV and DPV, respectively. The proposed method has accurately recovered pholcodine in spiked serum samples with a recovery percentage ranging from 1.2 to 2.9%. The optimized method is found to be accurate, precise, and robust by applying validation parameters provided by International Council for Harmonization. Two green metrics were computed to assess the method’s greenness, the findings showed that the developed method is environmentally friendly with minimum sample preparation steps.

**Supplementary Information:**

The online version contains supplementary material available at 10.1186/s13065-024-01146-x.

## Article highlights

1. Fabrication of electrochemical electrode using graphene nanoplatelets modified with polythiophene nanospheres.
2. Voltammetric analysis of pholcodine using square wave voltammetry and differential pulse voltammetry with method validation according to International Council for Harmonization (ICH) guidelines.
3. Assessment of the method’s greenness using two green assessment tools; analytical GREEness metric (AGREE) and complementary green analytical procedure index (ComplexGAPI) software tools.

**Supplementary Information:**

The online version contains supplementary material available at 10.1186/s13065-024-01146-x.

## Introduction

**Fig. 1:**
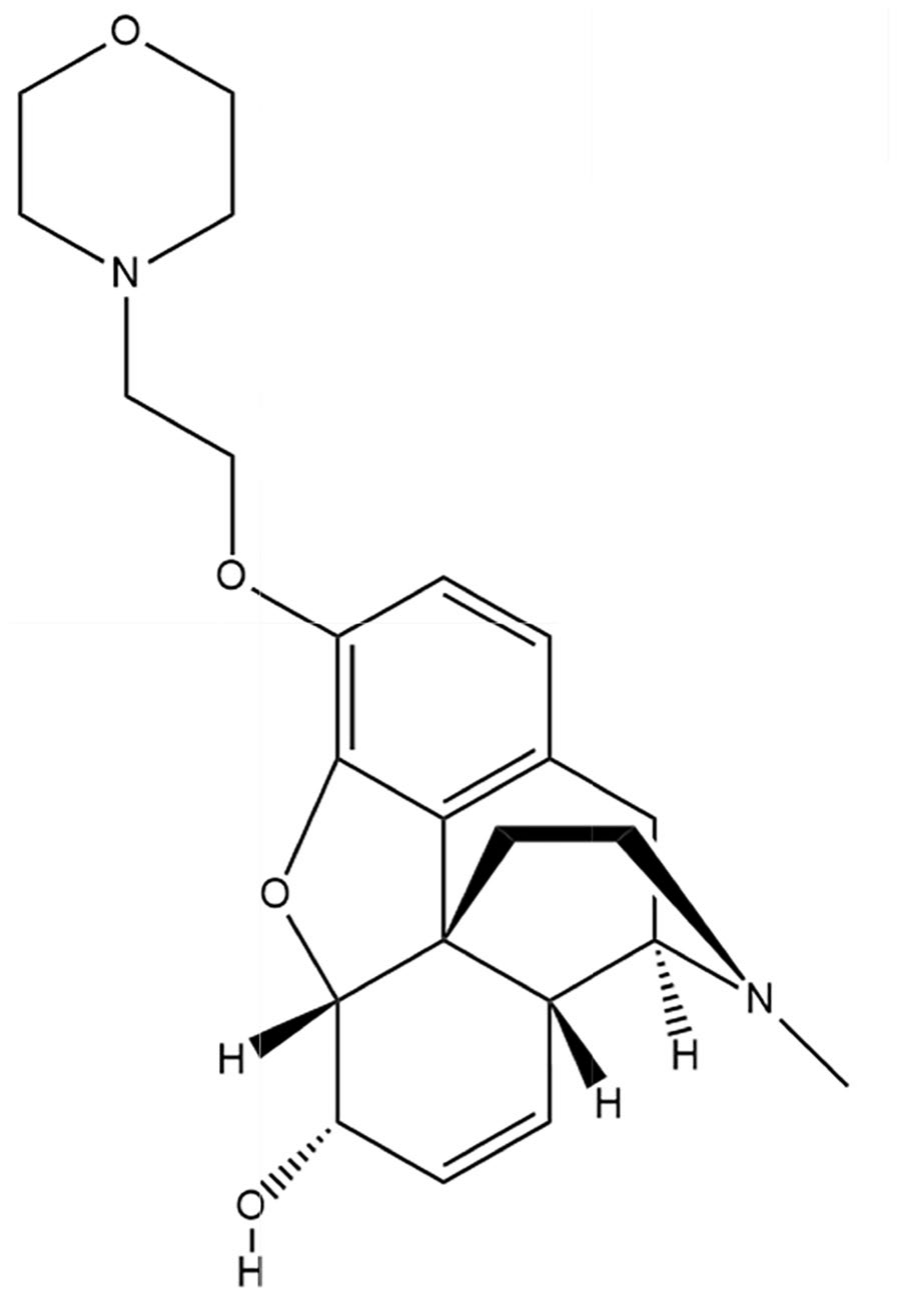
Structure of pholcodine

Pholcodine (PHL), a semi-synthetic opioid drug derivatized from codeine (Fig. 1) [1, 2], is a cough suppressant used to treat dry, unproductive coughs [3]. The main metabolites of PHL are Nor-P, desmorpholino-hydroxy-P, nor-desmorpholino-hydroxy-P with traces of morphine [4]. While PHL exhibits mild sedative behavior, it lacks the analgesic effects of morphine and codeine [2]. PHL has become a subject of concern in recent years, with Egypt reporting incidents of misuse and potential abuse associated with the drug. [5]. Several countries have taken action to restrict pholcodine (PHL) due to concerns about its safety. Notably, the Norwegian government banned its over-the-counter (OTC) sale in 2011 [6]. Later in 2022, the European Medicines Agency (EMA) [7], the British government [8], and the World Health Organization (WHO) [9] issued reports highlighting the potential risk of perioperative anaphylactic shock associated with PHL use (up to 12 months prior to surgery) in combination with neuromuscular blocking agents [10–12]. This newly identified risk called the "ALPHO case study" [13], emphasizes the potential for serious adverse reactions beyond what was previously known. As a result, several markets have taken further steps to restrict PHL, either fully banning its OTC sale or requiring a prescription [7–12]. Accordingly, pholcodine detection is crucial in forensic analysis and clinical therapeutic monitoring.

Pholcodine (PHL) has attracted significant interest. Numerous studies have employed various analytical techniques to quantify PHL, including UV/Vis spectrophotometry [14], spectrofluorimetry [15, 16], high-performance liquid chromatography (HPLC) [17–20] in presence of guaiacol [21, 22]. Other methods include gas chromatography (GC) [23], thin-layer chromatography [24], liquid chromatography electrospray ionization mass spectrometry (LC–ESI–MS) for impurity detection [25], chemiluminescence [26], and potentiometric analysis [5, 27] for accurate quantitative analysis of PHL. Notably, no reports exist on using electroanalytical voltammetry for PHL analysis. Voltammetric methods are known for their promptness, high sensitivity, selectivity, and cost-effectiveness [28–37]. Investigating the electrochemical behavior of PHL could therefore pave the way for a rapid and reliable analytical method, potentially laying the foundation for further forensic or bioanalytical applications.

This study fabricated an electrode using a graphene nanoplatelets paste. Graphene's large surface area makes it an ideal material for electrodes due to its excellent conductivity, electrical, and mechanical properties. Adding nanoparticles, such as metals or polymers, further enhances electron transfer and sensitivity [38–40]. Polythiophene (PTs) was chosen for this purpose due to its high conductivity [41] electrochemical stability [42], and sensitivity [43]. PTs enhance electron transfer [44], making them a candidate for electrode fabrications in chemical/biosensing [43–47], batteries, electro-polymerization [43], and capacitors. Therefore, PTs nanoparticles were incorporated with GNPs to improve the signal sensitivity of the fabricated electrode (GNPs/PTs).

The GNPs/PTs electrode characterization was performed using transmission electron microscopy (TEM), Fourier-transform infrared spectroscopy (FTIR), X-ray crystallography (XRD), X-ray photoelectron spectroscopy (XPS), and electrochemical impedance spectroscopy (EIS). Calculation of the active surface area and number of electron transfers were studied using cyclic voltammetry (CV) using potassium ferrocyanide (K_4_[Fe(CN)_6_]) in 0.1 M KCl as a redox probe. The electrochemical properties of PHL, effect of scan rate, and the effect of pH on GNPs/PTs electrode were investigated using cyclic voltammetry (CV), chronoamperometry, square wave voltammetry (SVW), and differential pulse voltammetry (DPV). Total validation of the SWV and DPV methods for PHL using the GNPs/PTs electrode was performed according to ICH guidelines Q2 (R1).

The greenness and environmental impact of the developed approach were evaluated using two metrics: the AGREE (Analytical GREEnness metric) tool and the ComplexGAPI (Complementary green analytical procedure index). The AGREE metric assesses a method's greenness based on twelve principles of green analytical chemistry. Each principle's weight can be changed for assured flexibility. Each sector of the clockwise figure illustrating the 12 principles is colored from red to yellow to green, signifying the degree of greenness associated with each concept in the methodology. A score closer to one indicates a greener approach. [48–51]. The ComplexGAPI green assessment is a sophisticated tool evaluating the environmental impact of analytical operations. ComplexGAPI evaluates all the procedure aspects, including sample collection, preparation, and analysis, as well as the synthesis and manufacturing of materials required for the method. The tool uses a pictogram made up of five pentagrams and a hexagon to symbolize each step of the process and how it affects the environment. The pentagrams are colored green, yellow, or red, accordingly, to denote a low, medium, or high influence. The hexagon turns green when certain requirements are met, such as using materials that are renewable or biodegradable. This tool aids in improving analytical chemistry's sustainability by tracking progress and identifying opportunities for adopting greener practices [52, 53].

The purpose of this study is the fabrication of GNPs/PTs electrode and developing a novel electrochemical approach for sensitive and green PHL detection with good reliability and reproducibility.

## Experimental

### Materials

Graphene, nanoplatelets (2–10 nm, 15,575,886), obtained from Acros Organics, Thermo Scientific Chemicals, US. Poly(3,4-ethylenedioxythiophene) nanoparticles dispersion in H_2_O, (675,288–25 mL) and human serum (H4522-20 mL) were obtained from Sigma Aldrich, Germany. PHL standard (purity, 99.2%) was generously provided from Amoun Pharmaceutical Co., Egypt. Potassium chloride (KCl) was obtained from TopChem pharmaceuticals, Ireland. Absolute ethanol, mineral oil, sodium phosphate dibasic heptahydrate, sodium phosphate monobasic monohydrate, phosphoric acid (H_3_PO_4_), sodium hydroxide (NaOH), and potassium ferrocyanide (K_4_[Fe(CN)_6_]) were obtained from Piochem, Giza, Egypt.. All Chemicals used in the experiments were of high analytical grade, sourced from various companies.

### Instrumentation

µStat-I 400s potentiostat/galvanostat/impedance analyzer (EIS), dropsens, Germany, was used to perform voltammetric measurements. The three-electrode system consists of a modified GNPs/PTs working electrode, an Ag/AgCl reference electrode in 3M KCl, and a platinum counter electrode. The potentiostat was operated by DropView 8400 software. pH adjustment was performed with a Jenway 3510 pH meter, UK. ATR-Fourier-transform infrared spectroscopy (FTIR), Alpha II (Brucker), Germany, was used to analyze the chemical composition of the electrode. Transmission electron microscopy (TEM): JEM-2100 Plus, (Electron Microscope, JEOL, Tokyo, Japan) was utilized to image the morphology of the electrode. X-ray diffractometer (XRD): Advance D8 diffractometer (Bruker), Germany, was used to identify the electrode’s crystalline phases. K-ALPHA (Thermo Fisher Scientific, USA) was used to obtain XPS data, characterized with a monochromatic X-ray Al K-alpha radiation source, a 400 mm spot size, and a 200 eV whole spectrum pass energy.

### Fabrication of the working electrode

The working electrode was fabricated by mixing GNPs powder (2–10 nm) with mineral oil in a ratio (70:30, w/w) using a mortar and pestle until a homogenous paste was formed. GNPs paste was mixed with PTs in ratio (8:2, w/v) and it was sonicated for 30 min, then placed in the oven for 1 h to obtain a firm paste. The modified paste was stored at room temperature in a well-sealed container. Prior to its use, it was firmly packed into a 3 mm empty working electrode (BAS Inc., Japan). The surface of the electrode was smoothened and polished using filter paper.

### Standard solution

A Stock solution of 1 g/L was prepared by dissolving PHL standard drug in absolute ethanol. Subsequent serial dilutions were prepared using PBS buffer containing 0.1 M KCl, across a concentration range of 10–45 mg/L. The PBS buffer (0.075 M sodium phosphate dibasic heptahydrate and 0.025 M sodium phosphate monobasic monohydrate) was adjusted to the required pH with 0.1 M NaOH and 0.1 M H_3_PO_4_.

### Characterization of the electrode electrochemical properties

Morphology characterization of GNPs/PTs electrode was performed using transmission electron microscope (TEM), JEM-2100 Plus, electron microscope, JEOL, Japan). To identify the chemical composition and crystalline phase of the electrode; FTIR, XRD, and XPS were used. For electrochemical characterization, cyclic voltammetry (CV), differential pulse voltammetry (DPV), square wave voltammetry (SWV), electrochemical impedance spectroscopy (EIS), and chronoamperometry were performed.

### Calibration curve, validation, and application

Calibration curves were constructed by plotting the peak heights, after baseline correction, of different PHL concentrations measured by DPV and SWV against standard PHL concentrations. Total validation of the developed approach was performed according to ICH (Q2) parameters.

For the detection of unknown PHL samples, the electrode potential was assessed using the standard addition method. The serum was initially diluted tenfold using PBS buffer (pH 7.4) and spiked with varying PHL concentrations (20, 30, and 40 mg/L). Afterward, the optimized SWV and DPV approach was used to measure the peak current (Ip) using the GNPs/PTs electrode. The found concentration was calculated using the regression equation derived from the calibration curve and the recorded Ip signal.

## Results and discussion

### Electrochemical behaviour of the modified electrode

**Fig. 2:**
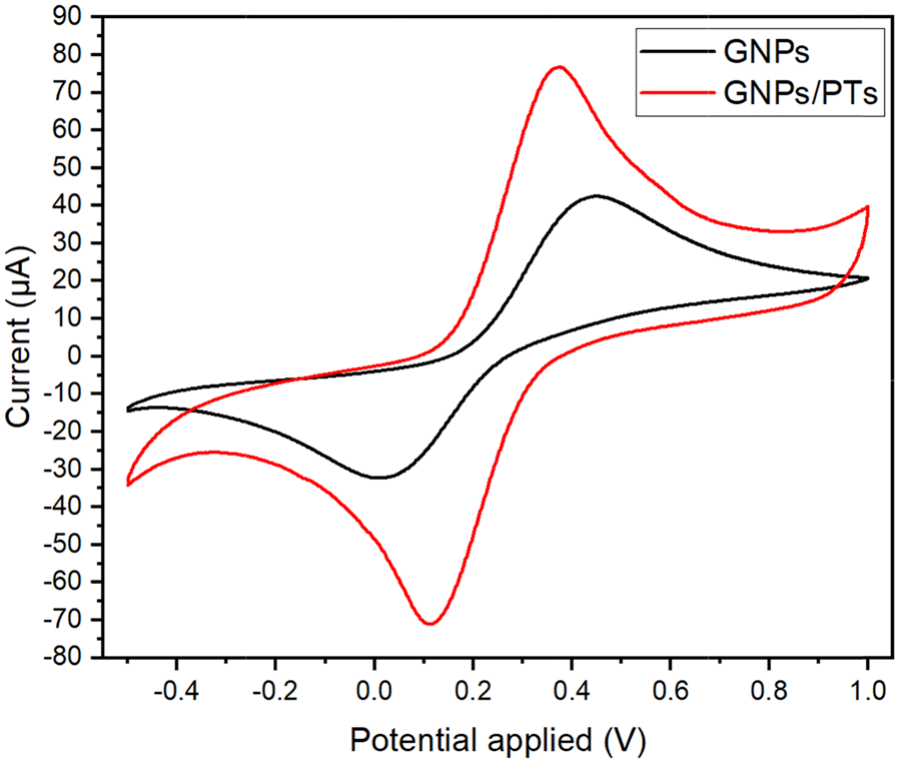
Cyclic voltammograms of 4.8 mM K_4_[Fe(CN)_6_] in 0.1 M KCl using GNPs electrode (black line) and GNPs/PTs electrode (red line), scan rate 75 mV/s, showing the effect of PTs modification on electron transfer efficiency

4.8 mM potassium ferrocyanide (K_4_[Fe(CN)_6_]) in 0.1 M KCl was used to evaluate the performance of modified electrodes as a redox probe. Cyclic voltammograms were recorded for each step of the electrode fabrication process. The peak-to-peak separation (∆E) and peak height were determined for each voltammogram. The potential was swept from + 1 to -0.4 V and swept back at a scan rate of 75 mV/s. Characteristic anodic and cathodic peaks for the redox probe were observed in the voltammograms as shown in Fig. 2. The ∆E values were 0.60 and 0.26 V for GNPs and GNPs/PTs respectively. The anodic peak heigh increased from 22.3 μA to 73.2 μA for GNPs and GNPs/PTs, respectively and the cathodic peak height from -16.8 µA to -58.8 µA for GNPs and GNPs/PTs, respectively. This represents approximately a threefold enhancement in the redox probe current. The enhanced electron transfer and conductivity of the modified electrode were reflected in the ∆E values, sharper peak, and higher peak heights compared to the unmodified GNPs electrode. The modifications to the electrode enhanced its electrochemical response, which will result in significant sensitivity and detection limit improvements for the target analytes.

### Electrode characterization

**Fig. 3:**
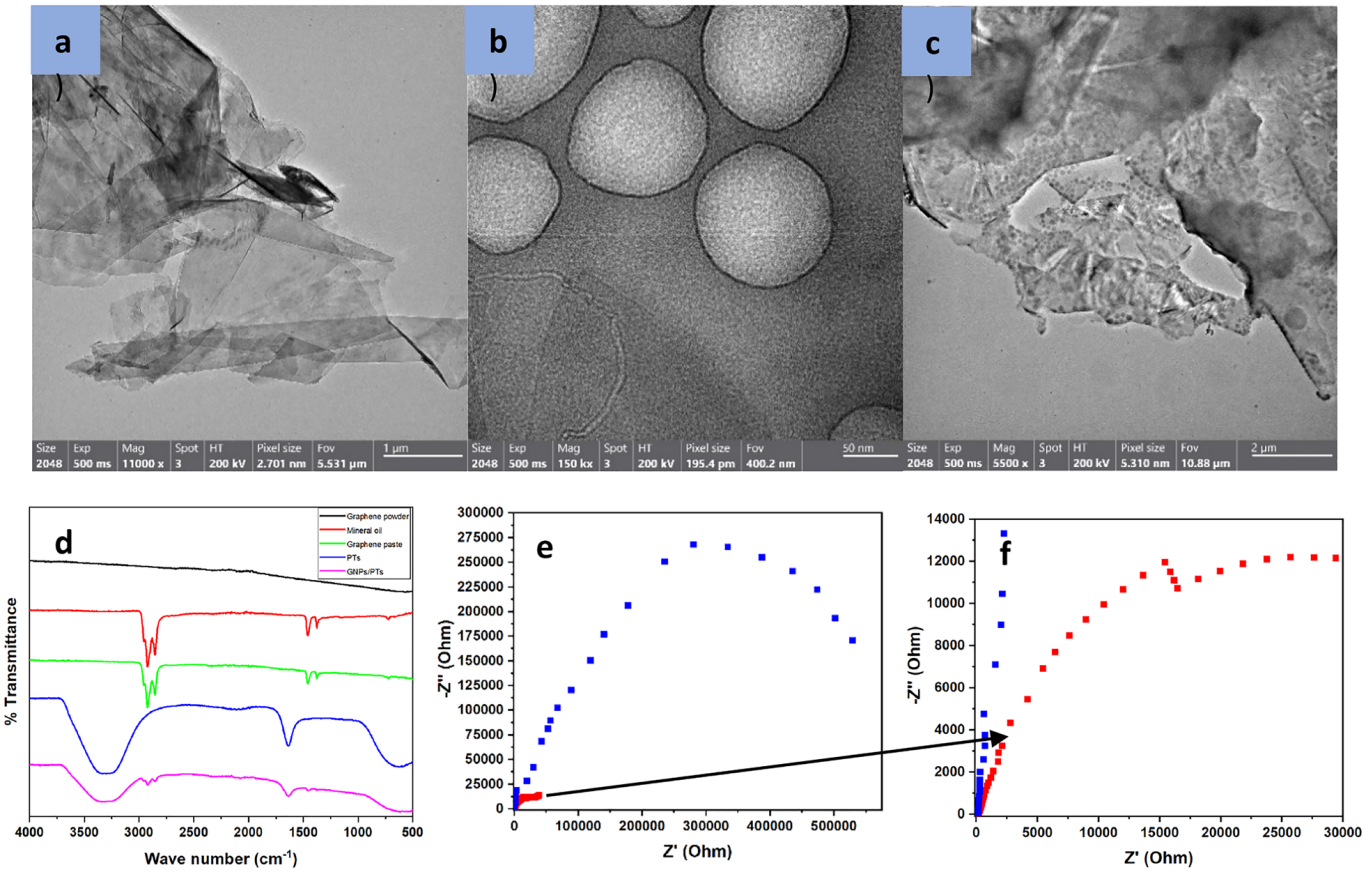
TEM images of **a** GNPs, **b** PTs, and **c** GNPs/PTs, **d** FTIR spectra of graphene powder (black line), mineral oil (red line), graphene paste (green line), polythiophene polymer (blue line), and graphene nanoplatelets mixed with polythiophene (purple line), **e** Nyquist plots of GNPs (blue dots) and GNPs/PTs electrode (red dots) using 5 mM K_4_[Fe(CN)_6_] in 0.1 M KCl as redox probe, and **f** Nyquist plots of GNPs/PTs electrode (red dots)

TEM images in Fig. 3 reveal that the GNPs (Fig. 3a) appear as flat sheets with sharp edges, while the PTs nanoparticles (Fig. 3b) are spherical in shape. Figure 3c demonstrates the incorporation of PTs nanospheres within the GNPs sheets.

FTIR spectra presented in Fig. 3d show the characteristic absorption bands of each component. GNPs alone do not exhibit any absorption bands. Meanwhile, mineral oil, used as a binder to paste the GNPs powder, showed peaks at 2917 & 2854 cm^−1^ which correspond to C–H stretching, and 1464 & 1379 cm^−1^, corresponding to C–H scissoring and methyl rock, respectively. Additionally, a peak at 722.7 cm^−1^ indicates C=C bending. The PTs absorption spectrum showed peaks at 1638 cm^−1^, characteristic of C=C, and 673.5 cm^−1^, corresponding to C–S thiophene. Eventually, the electrode paste spectrum integrates the functional groups observed in both the GNPs and PTs spectra.

Figure 3e and f present the Nyquist plots comparing the GNPs electrode and the GNPs/PTs electrode using 4.8 mM K_4_[Fe(CN)_6_] in 0.1 M KCl. Semifit calculations revealed an electron resistance of 236 Ω for GNPs electrode and 45.8 Ω for the GNPs/PTs electrode, confirming the observations from the cyclic voltammograms and the calculated activated surface area. The addition of PTs to GNPs decreased the resistance, leading to enhanced currents generated from the oxidation–reduction system.

X-ray diffraction analysis (Additional file 1: Figure S1) of GNPs revealed a characteristic diffraction peak at 2Ɵ = 26.6°, confirming the presence of graphene. Upon integrating PTs with GNPs, the intensity counts at this peak (26.6°) increased from 427 to 505. This increase in intensity indicates the successful incorporation of PTs with GNPs. XPS analysis was performed to validate the surface modification of GNPs with PTs, Additional file 1: Figure S2 presents the spectrum of GNPs/PTs electrode. The emergence of distinct peaks at 282.1 eV and 168.5 eV, attributable to C(1s) and S(1s), respectively, confirms the successful integration of PTs with GNPs.

### Electrode surface area

The effect of PTs modification on the electrode's voltammetric behavior using 4.8 mM ferrocyanide in 0.1 M KCl. Figure 2 illustrates cyclic voltammograms of unmodified GNPs and modified GNPs/PTs electrode. The voltammograms demonstrate that PTs enhance the peak height and sharpness, consistent with improved electron transfer on the electrode surface. The geometric surface area of the electrode was 0.071 cm^2^. The activated surface area of the electrode was calculated using Randles-Ševčík equation (Eq. 1), which describes the relationship between the peak current and the scan rate in a CV experiment. The activated surface area of the was 0.029 cm^2^ for GNPs and 0.098 cm^2^ for GNPs/PTs. The activated surface area represents the portion of the electrode surface that participates in electron transfer reactions. A higher activated surface area indicates greater electrochemical activity. The use of mineral oil was used as a liquid binder in the preparation of GNPs paste initially decreased the activated surface area. However, the addition of PTs increased the activated surface area by threefold. This enhancement implies that the GNPs/PTs modification improves the electrode's electrochemical activity and could lead to better performance in electrochemical applications.

1 $${\text{i}}_{pa} = { 2}.{69 } \times {1}0^{5} \times \, \left( {D^{{1}/{2}} n^{{3}/{2}} AC\upsilon^{{1}/{2}} } \right)$$

### Electrochemical behavior of PHL

**Fig. 4:**
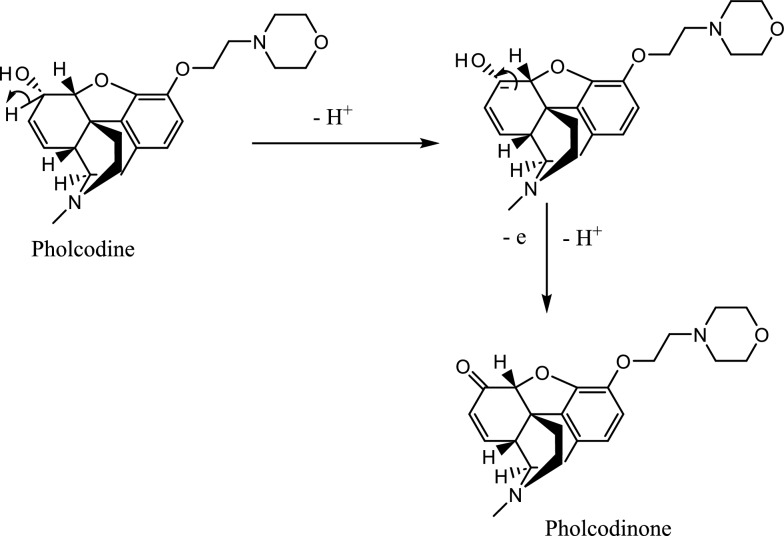
Proposed oxidation of pholcodine on GNPs/PTs electrode

The electrochemical properties of PHL have not been previously investigated using a voltammetric approach. CV of PHL using PBS in 0.1 M KCl at pH 7.4 were plotted as potential vs current by scanning potential from 0 V to + 1.3 V and swept back at a scan rate of 75 mV/s, the resulting voltammograms (Additional file 1: Figure S3) show a single anodic peak at 1.0 V with no corresponding cathodic peak, indicating an irreversible system. Two suggested mechanisms for PHL oxidation are proposed: dimerization similar to morphine and codeine [54, 55] and oxidation of the secondary alcohol in cyclohexene to a ketone [54]. The proposed mechanism that at the beginning C-H of the 2^ry^ alcohol breaks by oxidation, followed a formation of π bond between carbon and oxygen forming pholcodinone (Fig. 4). The proposed mechanism suggested losing one electron which was confirmed by the calculation of the number of electrons that participated in the reaction (Sect. "Effect of scan rate". Effect of scan rate). Also, to mention, potential required to oxidize PHL (Epa) ~ 1 V, compared to morphine Epa ~ 0.45 V, confirms that the oxidation of morphine was on the phenolic alcohol, followed by dimerization, which is easily oxidized and requires less potential. While the oxidation of secondary alcohol requires a higher potential to be oxidized to ketone.

### Effect of scan rate

**Fig. 5:**
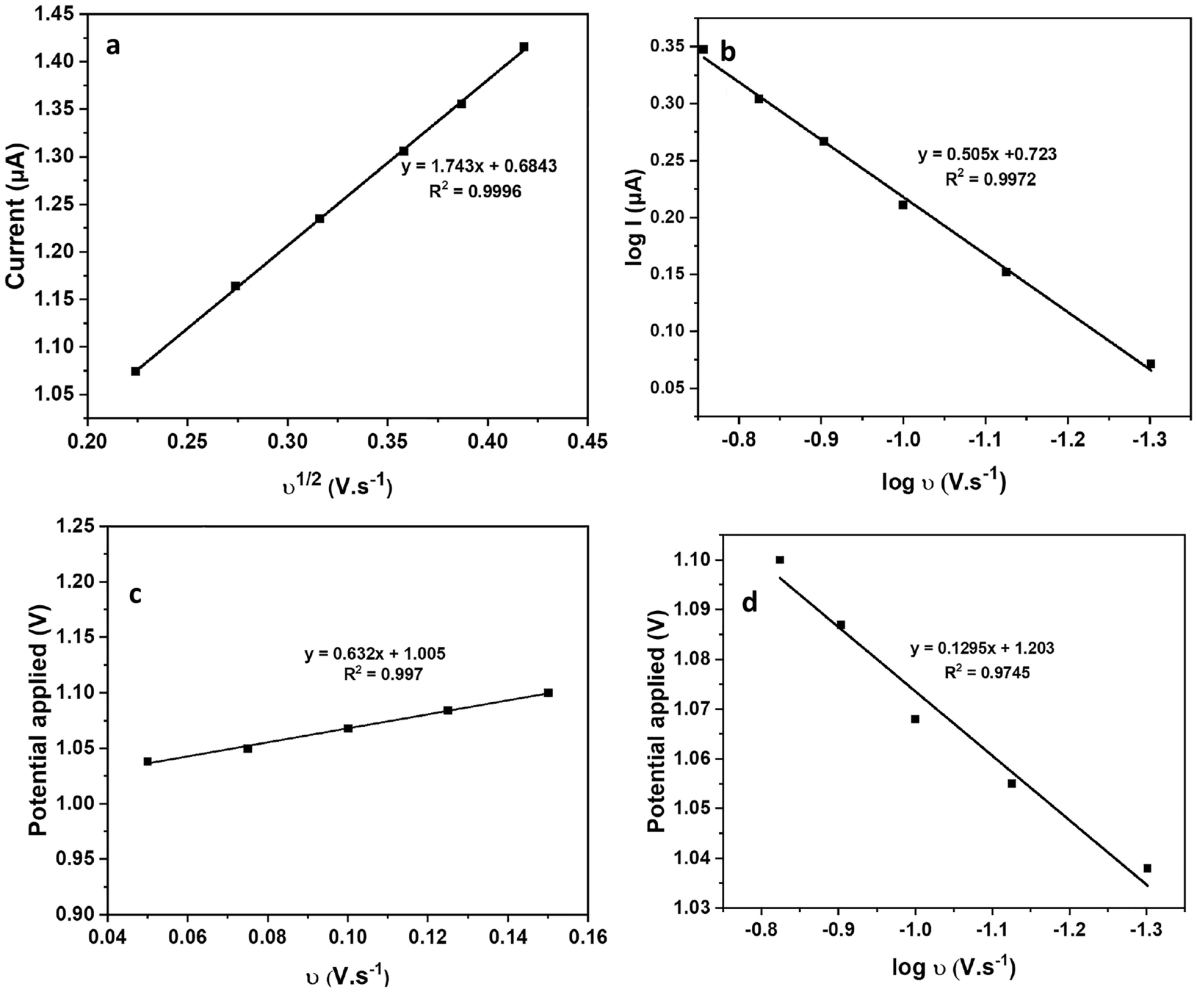
Effect of different scan rates (50, 75, 100, 125, 150, and 174 mV/s) by CV **a** current (µA) vs. SQR of scan rate, **b** current logarithm vs. scan rate logarithm, **c** potential applied vs. scan rate, and **d** potential applied vs. scan rate logarithm

The effect of scan rate was examined by applying cyclic voltammetry with a range of scan rates (50, 75, 100, 125, 150, and 175 mV.s^−1^) to determine the mechanism of PHL oxidation process. Illustrated in Fig. 5, a linear correlation between peak height (Ipa) and the square root of the scan rate, along with the linear correlation between the logarithm of the peak current (Ipa) and the logarithm of the scan rate (log υ), indicating that the system is controlled by diffusion.

2 $${\text{log I }}\left( {\mu {\text{A}}} \right) \, = \, 0.{5}0{\text{5 log }}\upsilon \, + \, 0.{723},{\text{ R}}^{2} = 0.{9972}$$

The slope of the linear regression equation (Eq. 2) was 0.505 which is close to the theoretical value of 0.5 for diffusion-controlled reactions. This further supports the conclusion that the PHL oxidation process is diffusion-controlled.

Plotting peak potential against the logarithm of the scan rate revealed that the oxidation peak potential shifted towards more positive values with increasing scan rate. This shift indicates that the reaction is irreversible.

Number of electrons involved in the reaction was calculated using Laviron's equation (Eq. 3):

3 $${\text{E }} = {\text{ E}}^{\text{o}} \left( {\frac{2.303RT}{{anF}}} \right) + {\text{log}}\left( {\frac{{{\text{RTK}}^{\text{o}} }}{anF}} \right) + \left( {\frac{{2.303{\text{RT}}}}{anF}} \right){\text{log}}\upsilon$$

The correlation between log υ and Ep can determine the αn from the obtained slope. The resulting slope was 0.1295 since the calculated value of α (irreversible system) was 0.505. Therefore, the calculated number of electrons was 1.1 ≈ 1 for each mol of PHL.

$${\text{E}}_{\text{p}} = \, 0.{\text{1295 log }}\upsilon \, + { 1}.{2}0{3}$$

### Effect of pH

The influence of different pH values on the behavior of 35 mg/L PHL on GNPs/PTs electrode using SWV. SWV measurements were conducted in PBS buffer with 0.1 M KCl at varying pH values ranging from 2 to 9. The pH was initially adjusted to 2 and then gradually increased to 9 using 0.1 M NaOH. As illustrated in Additional file 1: Figure S4, anodic peaks are absent in the voltammograms at pH values 2 to 5. Starting from pH 5, small anodic peaks emerge till pH 7, where a slight increase in height was observed till pH 9. Considering that blood pH typically falls within the range of 7.35 to 7.45, pH 7.4 was therefore chosen as the optimal condition for further voltammetric analysis.

### Chronoamperometry

**Fig. 6:**
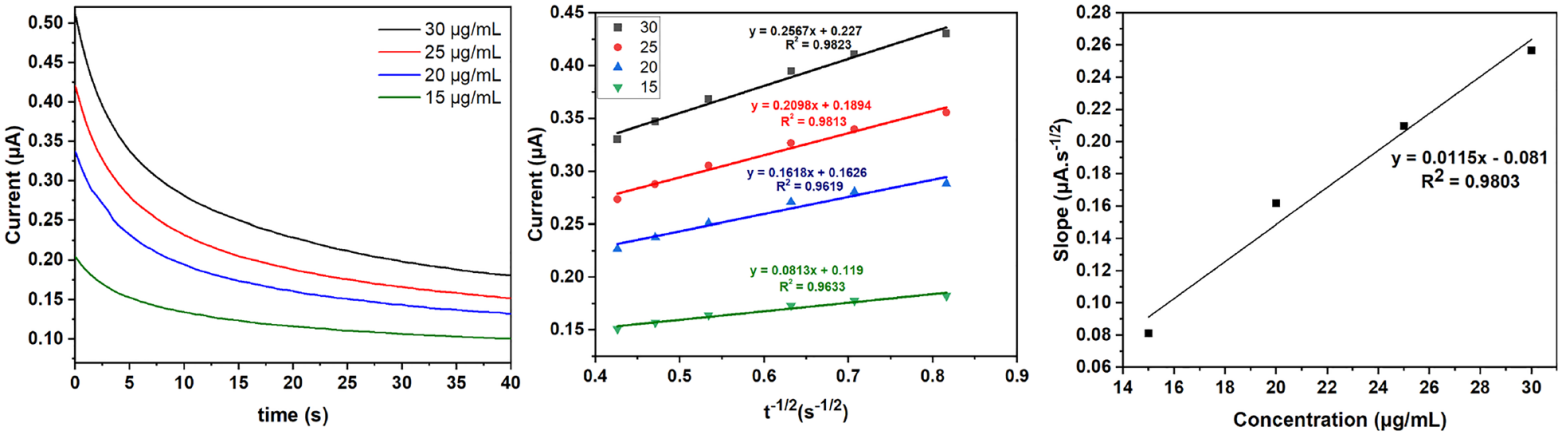
Chronoamperometry of four different concentrations of PHL at GNPs/PTs electrode surface

Determination of the diffusion coefficient (D) of PHL was done by measuring chronoamperometry of four different PHL concentrations. The chronoamperometric measurements were performed at a potential of 1 V for 40 s using the developed GNPs/PTs electrode. The current was plotted against the negative square root of the time (1.5, 2, 2.5, 3, 3.5, 4, 4.5 s^−1/2^), and the resulting slope for each equation was acquired. The calculated slopes were plotted against the four PHL concentrations for further calculation according to the Cottrell equation, A linear correlation resulted, and the slope calculated corresponded to D_PHL_. The calculated D_PHL_ was 1.15 × 10^–5^ cm/s as shown in Fig. 6, indicating a rapid diffusion of PHL on the modified electrode’s surface.

### Calibration curve and method validation

Different concentrations of KCl (0.05 and 0.1 M) as supporting electrolyte were examined and a sharper peak was observed with 0.1 M KCl. SWV variables were optimized to be 30 HZ, 3 mV, 4 mV for frequency, step potential, and amplitude potential, respectively to generate smooth and sharp peaks. The optimized DPV variables were 10 mV, 5 mV, 80 ms, and 50 mV/s for step potential, pulse potential, pulse time, and scan rate, respectively.

**Fig. 7:**
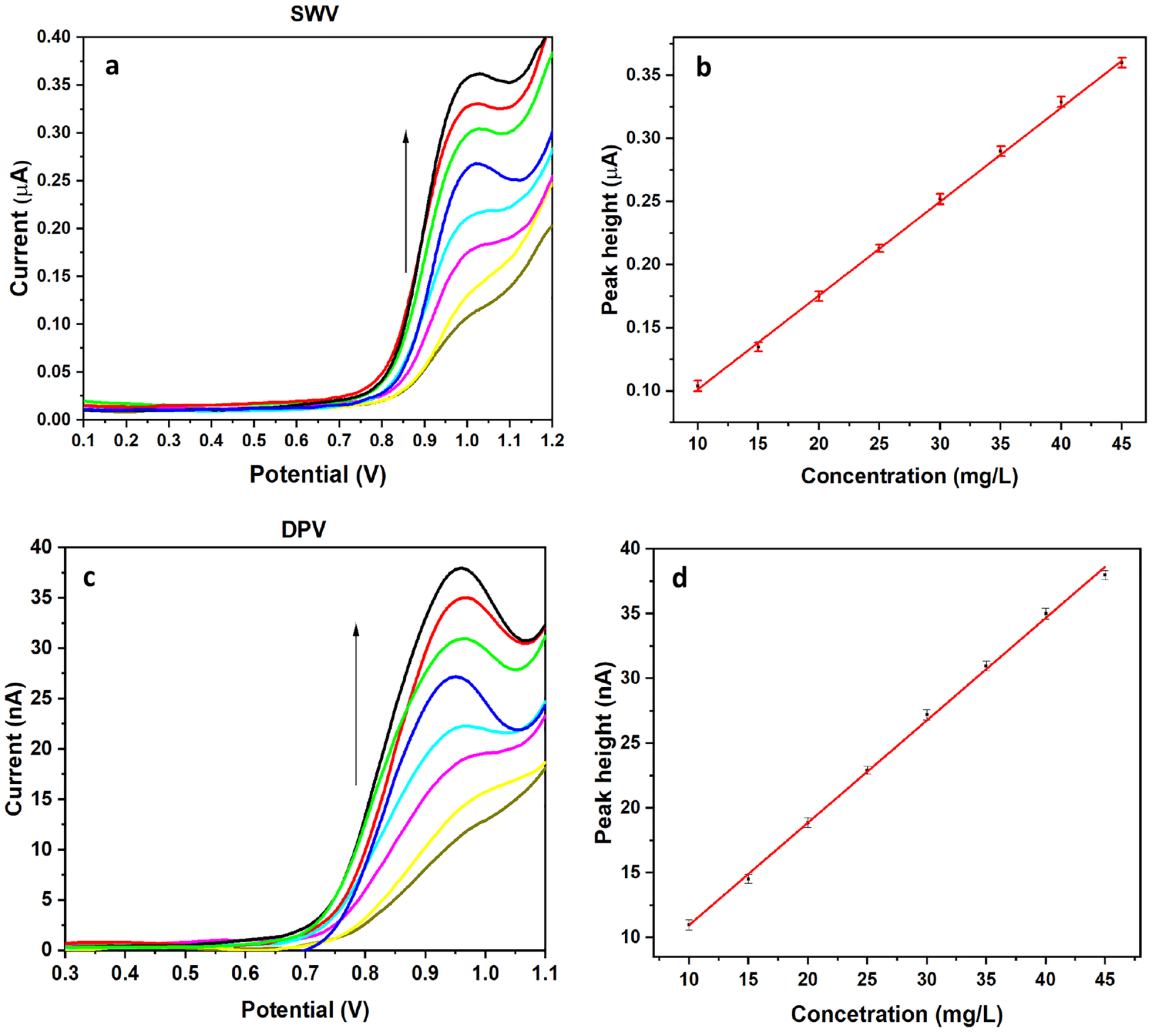
**a** SWV voltammograms of different concentrations (10–45 mg/L) of PHL, **b** SWV calibration curve of PHL. **c** DPV voltammograms of different concentrations (10–45 mg/L) of PHL, and **d** DPV calibration curve of PHL

Eight concentrations of PHL were used to construct a calibration curve with a linearity range from 10 to 45 mg/L. SWV and DPV approaches were performed to construct the calibration curves by plotting the current peak height (Ipa) against serial PHL concentrations (Fig. 7). The developed method was validated according to the ICH guidelines. The developed method showed a linearity ranging from 10 to 45 mg/L which reflects the reliability of this method for measuring PHL simply and accurately.

The limit of detection (LOD) and limit of quantification (LOQ) were calculated as 3.3 σ/S and 10 σ/S respectively, where σ is SD of intercept and S is the slope obtained from the regression equation computed from the calibration curve. As illustrated in Table 1. LOD of PHL was 1.41 and 1.51 mg/L for SWV and DPV approaches, respectively. While LOQ of PHL was 4.27 and 4.57 mg/L for SWV and DPV approaches, respectively. The presented approach has a remarkable sensitivity since there is no reported voltammetric method for PHL.

**Table 1:** ICH parameters results

ICH parameter	SWV	DPV
Linearity range	10–45 mg/L	10–45 mg/L
LOD (mg/L)	1.41	1.51
LOQ (mg/L)	4.27	4.57
(Correlation coefficient R^2^)	0.9993	0.9985
Slope	0.0074 ± 1.1 × 10^–4^	0.79 ± 0.012
Intercept	0.027 ± 0.0032	3.07 ± 0.36
Accuracy (mean ± SD)	99.45 ± 0.32	99.12 ± 0.56
Repeatability (%RSD)	0.62	0.67
Intermediate precision (%RSD)	0.79	0.84
Robustness (mean ± %RSD)	101.4 ± 1.1	102.1 ± 1.3

The accuracy of the proposed approach was carefully evaluated to ensure that PHL could be reliably and accurately quantified. The accuracy was determined by calculating the percentage recovery of the drug from three different concentrations (22, 32, and 42 mg/L) measured in triplicate. The mean ± SD of the three concentrations were then estimated from the %R, which is a measure of the percentage of PHL that is recovered, to assess the accuracy of the method. Table 1 shows the %R values obtained with PHL using both SWV and DPV methods, which demonstrates that the method is highly accurate, with a measurement of 99.45 ± 0.32 and 99.12 ± 0.56, respectively.

Precision is an important parameter for method evaluation. The repeatability and precision of the proposed method were thoroughly investigated. Repeatability, also known as intra-day precision, and intermediate precision, also known as inter-day precision, were assessed by analyzing three different samples in triplicate on a single day and across three different days, respectively. The relative standard deviation (%RSD) was used to quantify precision.

The intra-day precision results showed a %RSD of 0.62 and 0.67 for SWV and DPV, respectively, demonstrating the method's excellent repeatability within a single day. This means that the results obtained on the same day were very consistent. The inter-day precision results showed a %RSD of ± 0.79 and 0.84 for SWV and DPV, respectively, indicating the method's consistency and precision over multiple days. This means that the results obtained on different days were also very consistent. Both %RSD values were below 2%, which affirms the method’s high precision and reliability. Table 1 summarizes the precision assessment, including the %RSD values obtained for both intra-day and inter-day analyses. This further supports the method’s suitability for accurate and precise quantification of PHL.

The robustness of the proposed approach was carefully evaluated to ensure that it would be unaffected by minor changes in the experimental conditions. Two variables were deliberately altered within a small range: the pH value (7.4 ± 0.1) and the scan rate (75 ± 1 mV/s). The impact of these changes on the analytical results was carefully investigated, and the results are shown in Table 1. The results show that the voltammetric approach is robust, as the analytical results were not significantly affected by the changes in the experimental conditions.

### Reusability and stability of the fabricated electrode

To assess the reproducibility of the electrode, four electrodes were fabricated under identical conditions and their voltammetric responses were compared. The results showed that the electrodes were highly reproducible, with a %RSD of ± 0.36. This means that the electrodes produced very consistent results, even when they were fabricated at different times. The reusability of the electrode was also evaluated by comparing the voltammetric responses of an electrode that had been used before with those of an electrode that was newly fabricated. The two electrodes produced nearly identical responses (Additional file 1: Figure S5), indicating that the electrode can be reused without any significant loss in performance. The stability of the electrode was also examined over four weeks. The electrode responses remained stable over this time, further supporting the electrode's long-term performance. The electrode started to dry after 4 weeks, the addition of a few drops of mineral oil followed by repacking the electrode led to regaining its functionality as previously. The combined findings of the reproducibility, reusability, and stability assessments demonstrate that the developed electrode is robust and reliable. This makes it suitable for practical applications in analytical chemistry.

### Spiked samples in serum

Assessment of the electrode potential to detect unknown PHL samples was performed by standard addition. In the beginning, the serum was diluted tenfold and spiked with different concentrations of PHL (20, 30, and 40 mg/L). Concentrations of the spiked samples were evaluated using SWV and DPV developed methods. %Recovery and %RSD of each sample are illustrated in Table 2. The resulting measurements indicate the suitability and applicability of the electrode to measure unknown samples.

**Table 2:** Spiked serum samples recovery percentage and RSD for each approach (SWV and DPV)

Sample	Added conc. (mg/L)	Found conc. (mg/L)		%Recovery		%RSD ( n = 3)	
		SWV	DPV	SWV	DPV	SWV	DPV
Human Serum	20	20.07 19.26 19.39	19.92 19.14 19.39	96.52	97.42	2.2	2.0
	30	29.76 30.43 29.83	29.35 30.16 29.76	97.92	96.06	1.2	1.4
	40	36.62 37.83 38.37	36.49 38.64 37.29	97.14	96.95	2.3	2.9

### Greenness assessment of the method

The greenness assessment of the voltammetric approach was assessed using AGREE (analytical greenness metric) [49] and ComplexGAPI tools. For AGREE, twelve guidelines were used with a greenness calculator to create a graph that resembled a clock. The AGREE pictogram evaluates the impact on the environment from deep green to deep red, with a score displayed in the center. The AGREE pictograms for the developed SWV/DPV approach show green colors with scores of (0.88). The complex green analytical procedure index, or ComplexGAPI [52], has demonstrated that there is little chance of environmental harm from the suggested techniques. The results are shown using a color-coded pictogram with five pentagrams, which stand for sample preparation, reagent and solvent use, instrumentation, and a hexagon, which stands for pre-analysis condition. Green indicates an environmental impact that is significantly safer, yellow indicates troublesome impact, and red indicates an impact that is risky and should be avoided. This developed method produced 15 green colors and two yellow colors. While the hexagonal with 1.00 E-factor was all green. This method, depending on the green assessment results can be considered a green method and by comparison with the reported methods, it is greener as shown in Table 3.

**Table 3:** Greenness profile and comparative study of the developed method with previously reported methods

	This method	Reported method 1 [16]	Reported method 2 [22]
Technique	DPV and SWV	Fluorimetry	UPLC
Linearity	10–45 mg/L	0.05–6.0 mg/L	50– 1000 mg/L
LOD (mg/L)	1.41 (SWV) 1.51 (DPV)	0.01	NA
LOQ (mg/L)	4.27 (SWV) 4.57 (DPV)	0.02	NA
AGREE			
ComplexGAPI			

## Conclusion

In this study, we developed an enhanced electron transfer electrode by leveraging the combined effects of nanoparticles. Graphene nanoplatelets acted as a conductive scaffold to facilitate electron transfer, while polythiophene polymer increased the activated surface area and electron transfer for improved analyte interaction. The modified electrode was comprehensively characterized using TEM, FTIR, XRD, XPS, and EIS, confirming the successful integration of the nanoparticles. This tailored electrode demonstrably exhibited enhanced detection capabilities.

Our primary target was PHL, an antitussive medication previously withdrawn from the over-the-counter market in several countries due to safety concerns and reported abuse. Since the voltammetric analysis of PHL had not been reported before, we employed a comprehensive suite of techniques including CV, SWV, DPV, and chronoamperometric measurements. The developed method was validated based on ICH parameters, demonstrating excellent linearity between 10 and 45 mg/L, as well as sensitive limits of detection of 1.41 and 1.51 mg/mL for SWV and DPV, respectively and quantification of 4.27 and 4.57 mg/L for SWV. Furthermore, the method proved to be accurate, precise, and robust and detection of spiked samples in human serum with %RSD < 2, indicating reliable performance. Notably, the electrode displayed excellent reproducibility and robustness over four weeks. This highly sensitive, reliable, and green approach holds significant promise for a range of applications, including forensic analysis and clinical monitoring of pholcodine.

### Supplementary Information

**Additional file 1.** Additional figures.

## Data Availability

Most data generated or analyzed during this study are included in this published.

## References

[1] *National Center for Biotechnology Information (2023). PubChem Compound Summary for CID 86278131, Pholcodine monohydrate. Retrieved August 19, 2023 from *https://pubchem.ncbi.nlm.nih.gov/compound/Pholcodine-monohydrate*.* .

[2] Findlay JWA (1988). Pholcodine. J Clin Pharm Ther.

[3] Wisher D (2012). Martindale: the complete drug reference. J Med Libr Assoc..

[4] Maurer HH, Fritz CF (1990). Metabolism of pholcodine in man. Arzneimittelforschung.

[5] Abd-Rabboh HSM (2021). All-solid-state potentiometric ion-sensors based on tailored imprinted polymers for pholcodine determination. Polymers.

[6] Florvaag E (2011). IgE-sensitization to the cough suppressant pholcodine and the effects of its withdrawal from the Norwegian market. Allergy.

[7] https://www.ema.europa.eu/en/medicines/human/referrals/pholcodine-containing-medicinal-products.

[8] Mahase E (2023). UK withdraws pholcodine-containing cough and cold medicines over anaphylaxis risk in surgery. BMJ.

[9] https://www.who.int/news/item/31-03-2023-pholcodine-containing-remedies-anaphylactic-reactions.

[10] Florvaag E, Johansson SGO (2009). The Pholcodine Story. Immunol Allergy Clin North Am.

[11] Johansson SGO (2010). National pholcodine consumption and prevalence of IgE-sensitization: a multicentre study. Allergy.

[12] Brusch AM (2014). Exploring the link between pholcodine exposure and neuromuscular blocking agent anaphylaxis. Br J Clin Pharmacol.

[13] Mertes PM (2023). Pholcodine exposure increases the risk of perioperative anaphylaxis to neuromuscular blocking agents: the ALPHO case-control study. Br J Anaesth.

[14] Moustafa AA (2019). Novel Approach for the Simultaneous Determination of Carbinoxamine Maleate, Pholcodine, and Ephedrine Hydrochloride Without Interference from Coloring Matter in an Antitussive Preparation Using Smart Spectrophotometric Methods. J AOAC Int.

[15] Thomas AD (1975). Spectrophotofluorometric determination of some alkaloids containing a tertiary amine group. Talanta.

[16] Elmansi H, Belal F, Magdy G (2022). Determination of pholcodine alone or in combination with ephedrine in human plasma using fluorescence spectroscopy. Sci Rep.

[17] Denk OM, Watson DG, Skellern GG (2000). Micellar electrokinetic and high-performance liquid chromatographic determination of potential manufacturing impurities in pholcodine. J Chromatogr A.

[18] Petkovska A, Babunovska H, Stefova M (2011). Fast and selective HPLC-DAD method for determination of pholcodine and related substances. Macedonian J Chem Chem Eng.

[19] Chen ZR (1988). Determination of pholcodine in biological fluids by high-performance liquid chromatography with fluorescence detection. J Chromatogr B Biomed Sci Appl.

[20] Johansen M, Tønnesen F, Rasmussen KE (1992). Column-switching high-performance liquid chromatographic detection of pholcodine and its metabolites in urine with fluorescence and electrochemical detection. J Chromatogr B Biomed Sci Appl.

[21] Mansour FR, Khairy MA (2019). Stability Indicating RP-HPLC Method for Simultaneous Determination of Pholcodine and Guaiacol in Pharmaceutical Syrup. Pharm Chem J.

[22] Mohamed HM (2023). Exploiting the power of UPLC in separation and simultaneous determination of pholcodine, guaiacol along with three specified guaiacol impurities. BMC Chem.

[23] Johansen M, Rasmussen KE, Christophersen AS (1990). Determination of pholcodine and its metabolites in urine by capillary gas chromatography. J Chromatogr B Biomed Sci Appl.

[24] Denk OM (2010). Isolation and Identification of Three Potential Impurities of Pholcodine Bulk Drug Substance. J Pharm Pharmacol.

[25] Denk OM, Skellern GG, Watson DG (2002). Impurity profiling of pholcodine by liquid chromatography electrospray ionization mass spectrometry (LC-ESI-MS). J Pharm Pharmacol.

[26] Delouei NJ, Mokhtari A, Jamali MR (2017). Determination of pholcodine in syrups and human plasma using the chemiluminescence system of tris(1,10 phenanthroline)ruthenium(II) and acidic Ce(IV). Luminescence.

[27] Abd-Rabboh HSM (2021). Paper-based potentiometric sensing devices modified with chemically reduced graphene oxide (CRGO) for trace level determination of pholcodine (opiate derivative drug). RSC Adv.

[28] Scholz F (2015). Voltammetric techniques of analysis: the essentials. ChemTexts.

[29] Harsha, D. and S. Chetna, *Recent Advances in Voltammetric Sensing*, in *Frontiers in Voltammetry*, R. Shashanka, et al., Editors. 2022, IntechOpen: Rijeka. p. Ch. 5.

[30] Eid SM (2023). An innovative nanoparticle-modified carbon paste sensor for ultrasensitive detection of lignocaine and its extremely carcinogenic metabolite residues in bovine food samples: Application of NEMI, ESA, AGREE, ComplexGAPI, and RGB12 algorithms. Food Chem.

[31] Abdelshafi NA (2019). Microfluidic electrochemical immunosensor for the trace analysis of cocaine in water and body fluids. Drug Test Anal.

[32] Ghoreishi SM (2017). Application of experimental design for quantification and voltammetric studies of sulfapyridine based on a nanostructure electrochemical sensor. Arab J Chem.

[33] Ghoreishi SM (2015). Fabrication of a nickel titanate nanoceramic modified electrode for electrochemical studies and detection of salicylic acid. J Mol Liq.

[34] Khoobi A, Ghoreishi SM, Behpour M (2014). Sensitive and selective determination of hydroxychloroquine in the presence of uric acid using a new nanostructure self-assembled monolayer modified electrode: optimization by multivariate data analysis. Analyst.

[35] Khoobi A (2019). A sensitive lead titanate nano-structured sensor for electrochemical determination of pentoxifylline drug in real samples. J Nanostruct Chem.

[36] Foroughi MM (2014). Electrochemical determination of N-acetylcysteine and folic acid in pharmaceutical and biological samples using a modified carbon nanotube paste electrode. Int J Electrochem Sci.

[37] Mollaei M, Ghoreishi SM, Khoobi A (2019). Electrochemical investigation of a novel surfactant for sensitive detection of folic acid in pharmaceutical and biological samples by multivariate optimization. Measurement.

[38] Saki A, Pourghobadi Z, Derikvand Z (2022). Gold nanoparticles decorated with multi-walled carbon nanotubes/graphene oxide for voltammetric determination of dopamine in the presence of acetaminophen. J Electrochem Soc.

[39] Mani S (2015). Anti-tuberculosis drug pyrazinamide determination at multiwalled carbon nanotubes/graphene oxide hybrid composite fabricated electrode. Int J Electrochem Sci.

[40] Peik-See T (2014). Simultaneous electrochemical detection of dopamine and ascorbic acid using an iron oxide/reduced graphene oxide modified glassy carbon electrode. Sensors.

[41] Pascariu P (2019). Tuning electrical properties of polythiophene/nickel nanocomposites via fabrication. Mater Des.

[42] Inamuddin A, Alamry. KA *Application of Electrically Conducting Nanocomposite Material Polythiophene@NiO/Frt/GOx as anode for enzymatic biofuel cells.* Materials, 2020. **13**: 8. Doi: 10.3390/ma13081823. 10.3390/ma13081823 PMC7215782 32290640

[43] Si P (2007). Functional polythiophene nanoparticles: size-controlled electropolymerization and ion selective response. J Am Chem Soc.

[44] Pilo MI (2022). Poly(Thiophene)/graphene oxide-modified electrodes for amperometric glucose biosensing. Nanomaterials (Basel).

[45] Liu C (2007). Covalent immobilization of glucose oxidase on films prepared by electrochemical copolymerization of 3-methylthiophene and thiophene-3-acetic acid for amperometric sensing of glucose: effects of polymerization conditions on sensing properties. Eur Polymer J.

[46] Saljooqi A, Shamspur T, Mostafavi A (2020). The electrochemical sensor based on graphene oxide nanosheets decorated by gold nanoparticles and polythiophene for nicotine sensing in biological samples and cigarette. J Mater Sci.

[47] Al-Refai HH, Ganash AA, Hussein MA (2021). Polythiophene and its derivatives –based nanocomposites in electrochemical sensing: a mini review. Materials Today Communications.

[48] Wojnowski W (2022). AGREEprep – Analytical greenness metric for sample preparation. TrAC, Trends Anal Chem.

[49] Pena-Pereira F, Wojnowski W, Tobiszewski M (2020). AGREE—analytical GREEnness metric approach and software. Anal Chem.

[50] Pena-Pereira F (2022). A tutorial on AGREEprep an analytical greenness metric for sample preparation. Adv Sample Prep.

[51] Attia KAM (2023). Simultaneous analysis of the of levamisole with triclabendazole in pharmaceuticals through developing TLC and HPLC–PDA chromatographic techniques and their greenness assessment using GAPI and AGREE methods. BMC Chem.

[52] Płotka-Wasylka J, Wojnowski W (2021). Complementary green analytical procedure index (ComplexGAPI) and software. Green Chem.

[53] Płotka-Wasylka J (2018). A new tool for the evaluation of the analytical procedure: Green Analytical Procedure Index. Talanta.

[54] Garrido JMPJ (2004). Voltammetric oxidation of drugs of abuse II. Codeine Metab Electroanalysis.

[55] Garrido JMPJ (2004). Voltammetric oxidation of drugs of abuse I. Morphine Metab Electroanalysis.

