# Supplementary material for: Voltammetric analysis of pholcodine on graphene-modified GNPs/PTs with green assessment
Source: BMC Chem. 2024 Mar 6;18(1):48. doi: 10.1186/s13065-024-01146-x (PMC10919016; doi:10.1186/s13065-024-01146-x)
Supplement: Supplementary file 1 — Additional file 1. Additional figures. [file 13065_2024_1146_MOESM1_ESM.docx]

**Supplementary Figures**


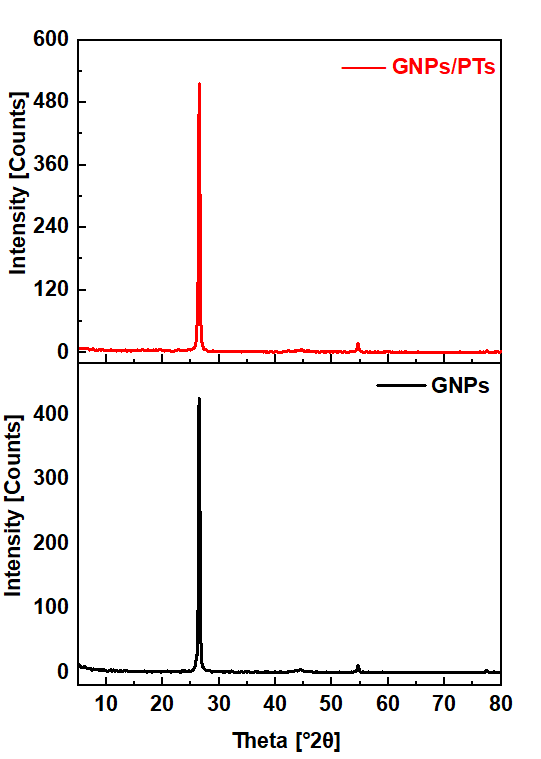


Figure S1: XRD of GNPs and GNPs/PTs


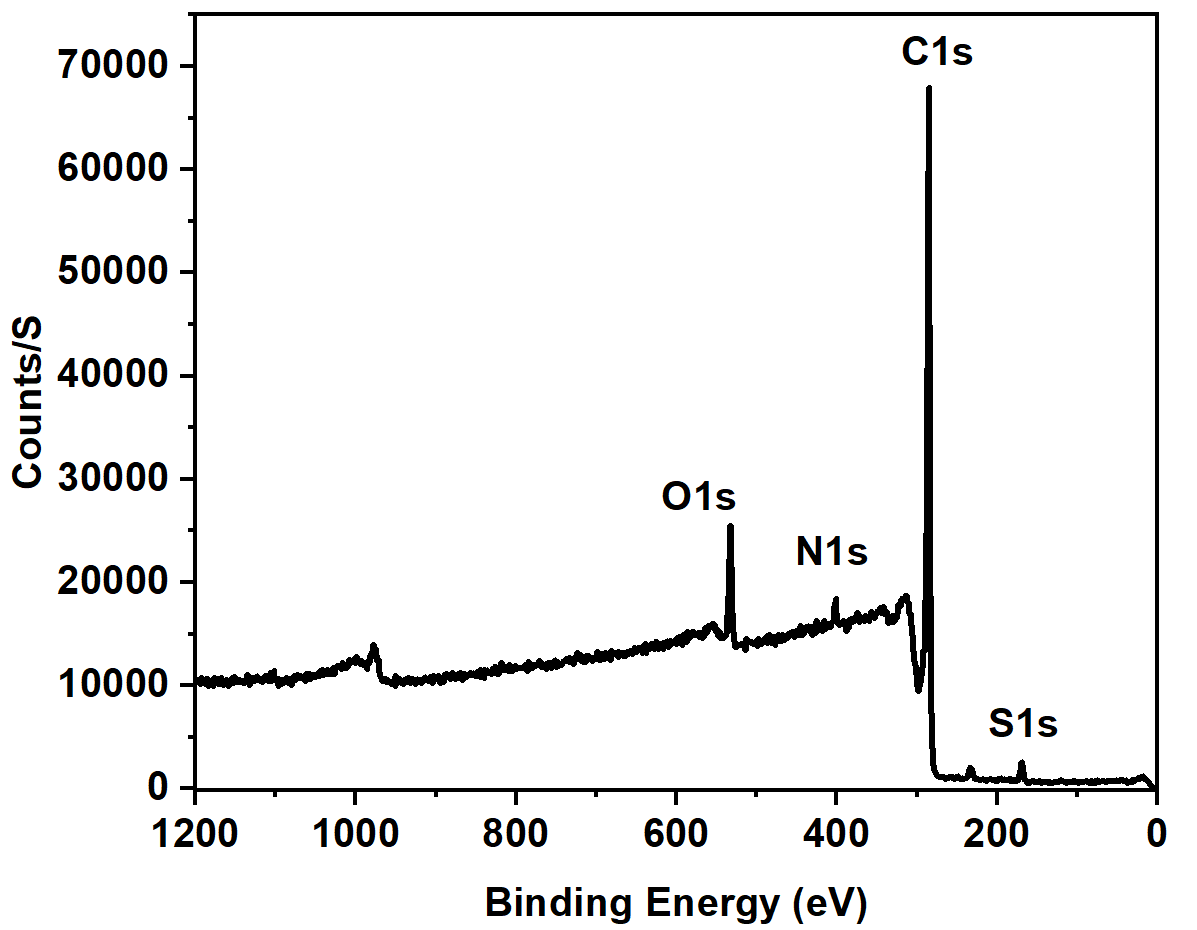


Figure S2: XPS of GNPs/PTs


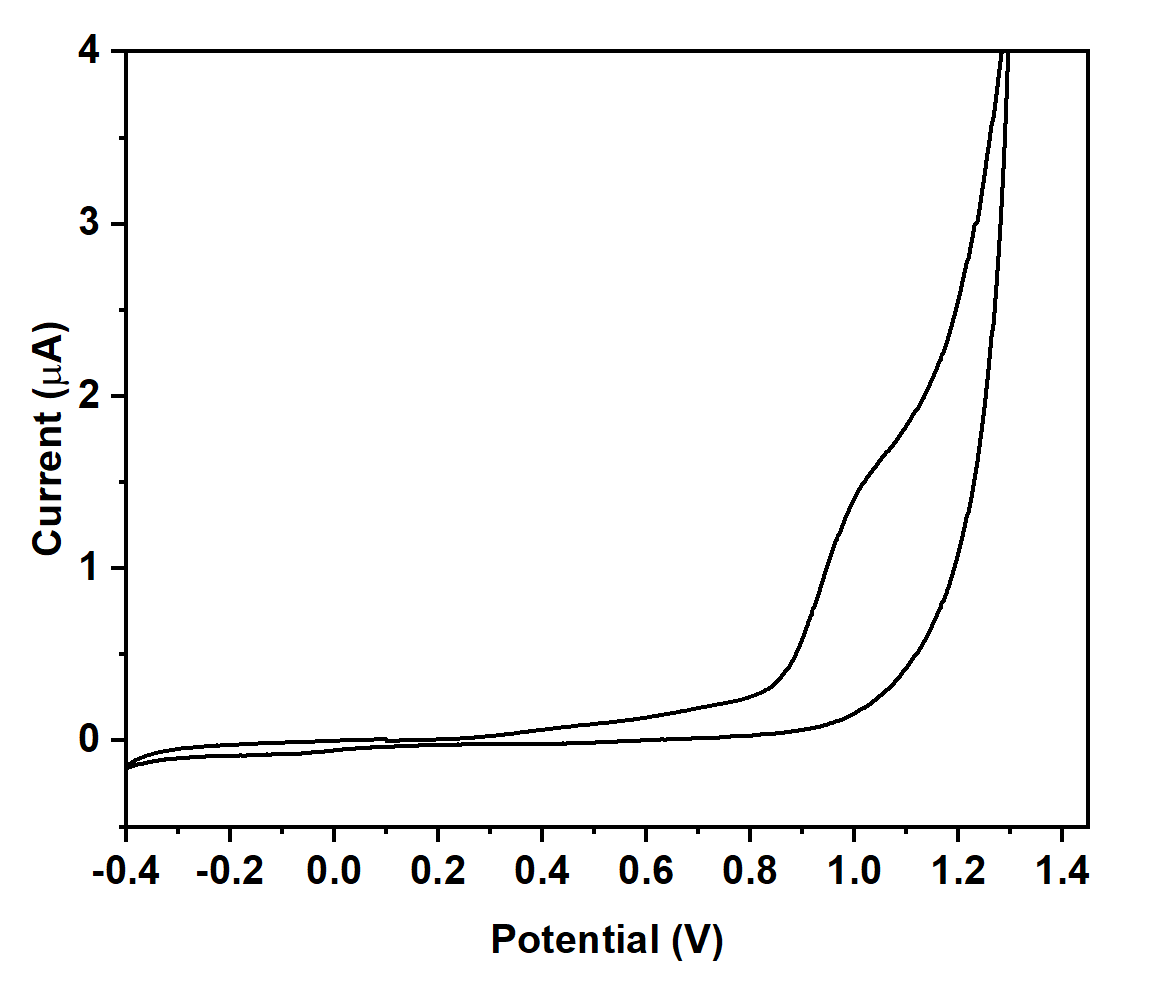


Figure S3: CV of PHL, showing irreversibility of PHL redox reaction


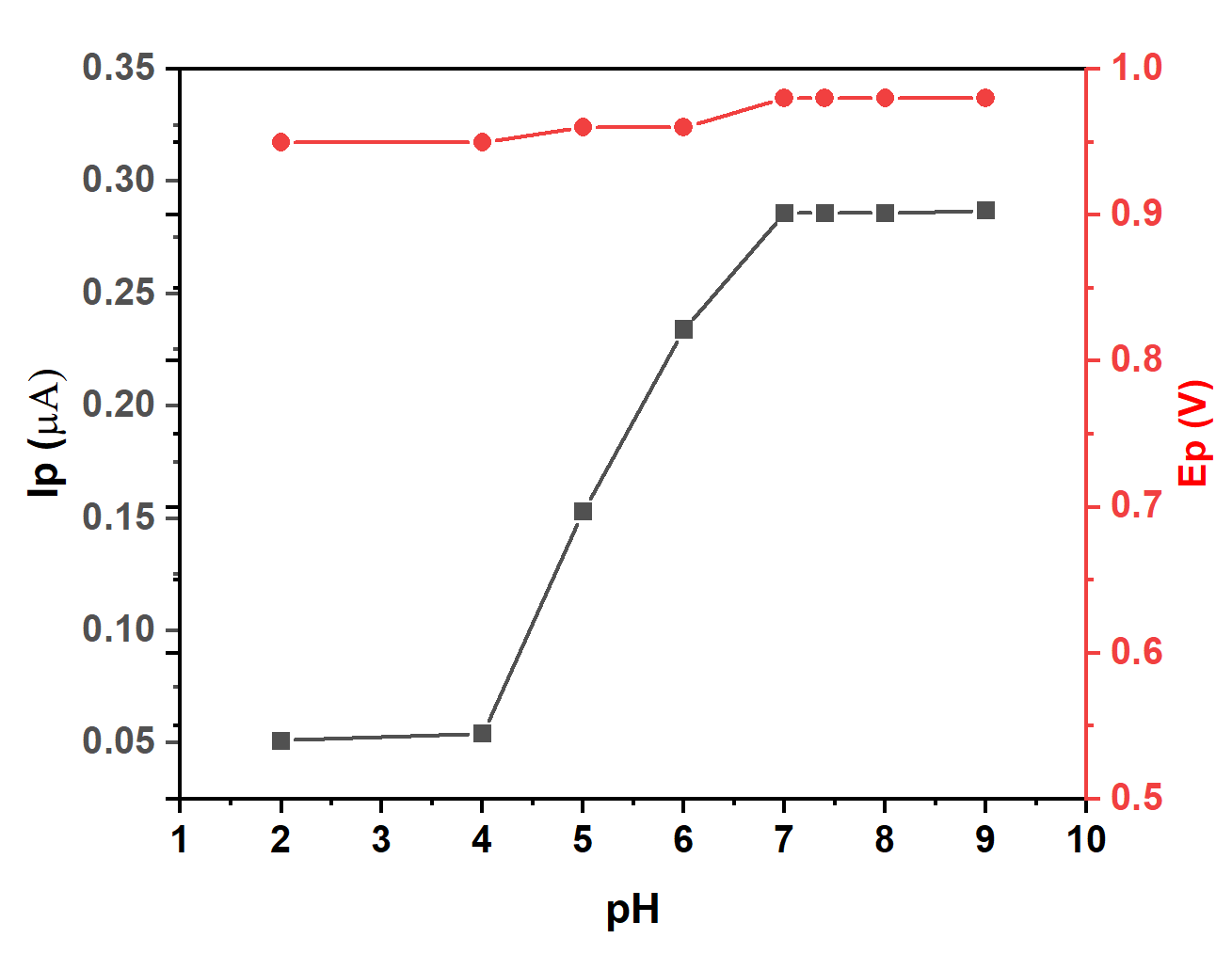
 Figure S4: The effect of pH on the peak current (square symbol) and peak potential (circle symbol) of PHL for SWV responses of 35 mg/L of PHL using GNPs/PTs electrode.

Figure S5: Reproducibility of GNPs/PTs electrode in PBS buffer in 0.1 M KCl (pH 7.6), containing 35 mg/L PHL.
